# Supplementary material for: Using Porcine Jejunum Ex Vivo to Study Absorption and Biotransformation of Natural Products in Plant Extracts: Pueraria lobata as a Case Study
Source: Metabolites. 2021 Aug 14;11(8):541. doi: 10.3390/metabo11080541 (PMC8398828; doi:10.3390/metabo11080541)
Supplement: Supplementary file 1 [file metabolites-11-00541-s001.zip › metabolites-1312546-supplementary.pdf]

Supplementary Material

Using porcine jejunum *ex vivo* to study absorption and biotransformation of natural products in plant extracts: *Pueraria lobata* as a case study

Joëlle Houriet[1, 2], Yvonne E. Arnold[1, 2], Léonie Pellissier[1,2,] Yogeshvar N. Kalia[1, 2] and Jean-Luc Wolfender[1, 2*]

[1] School of Pharmaceutical Sciences, University of Geneva, CMU, rue Michel-Servet 1, 1211 Geneva, Switzerland
[2] Institute of Pharmaceutical Sciences of Western Switzerland, University of Geneva, CMU, rue Michel-Servet 1, 1211 Geneva 4, Switzerland
* Correspondence: jean-luc.wolfender@unige.ch

Table of Contents

1 Feature based molecular network of PLRE 2
2 Semi-quantitative peak ratios from UHPLC-HRMS data processing 5
3 Quantitative results for PLRE 9
4 Quantitative results for puerarin and daidzin standard 11
5 Data processing of the metabolite profiling 12
6 Acquisition of the untargeted UHPLC-HRMS/MS metabolite profiling of PLRE 13
7 MZmine parameters for peak picking 14
8 Acquisition of the quantitative analyses 15

1 Feature based molecular network of PLRE

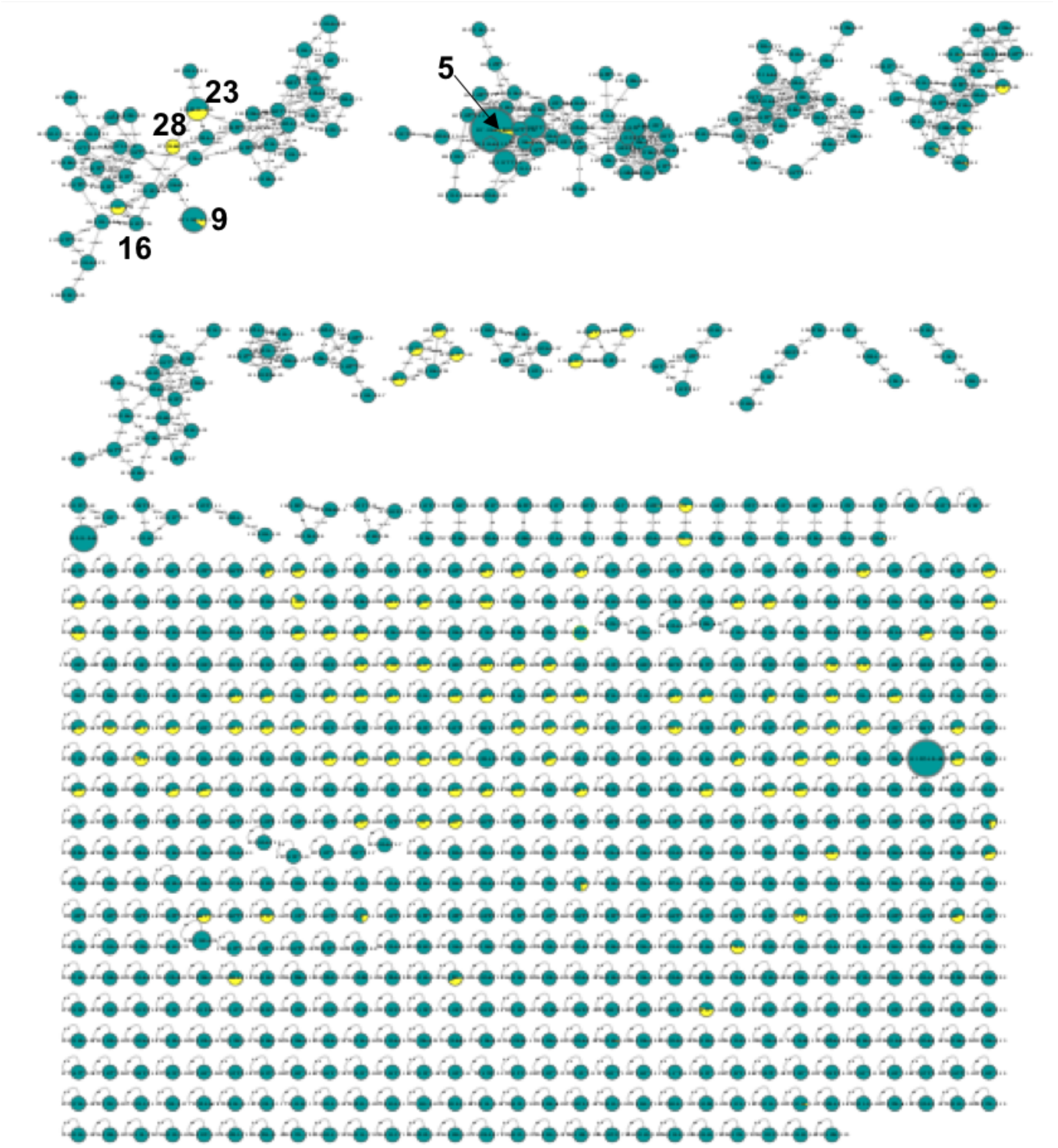

Supplementary Figure S1. Feature-based molecular network (FBMN) of the freeze-dried decoction of *Pueraria lobata* roots (PLRE) at 5 mg/mL, resulting from the data processing of UHPLC-HRMS/MS analyses (section 4.8). In blue-green, peaks detected in PLRE, in yellow, peaks detected in the solution of five standards. The numbers highlight the peaks of the five standards (table 1).

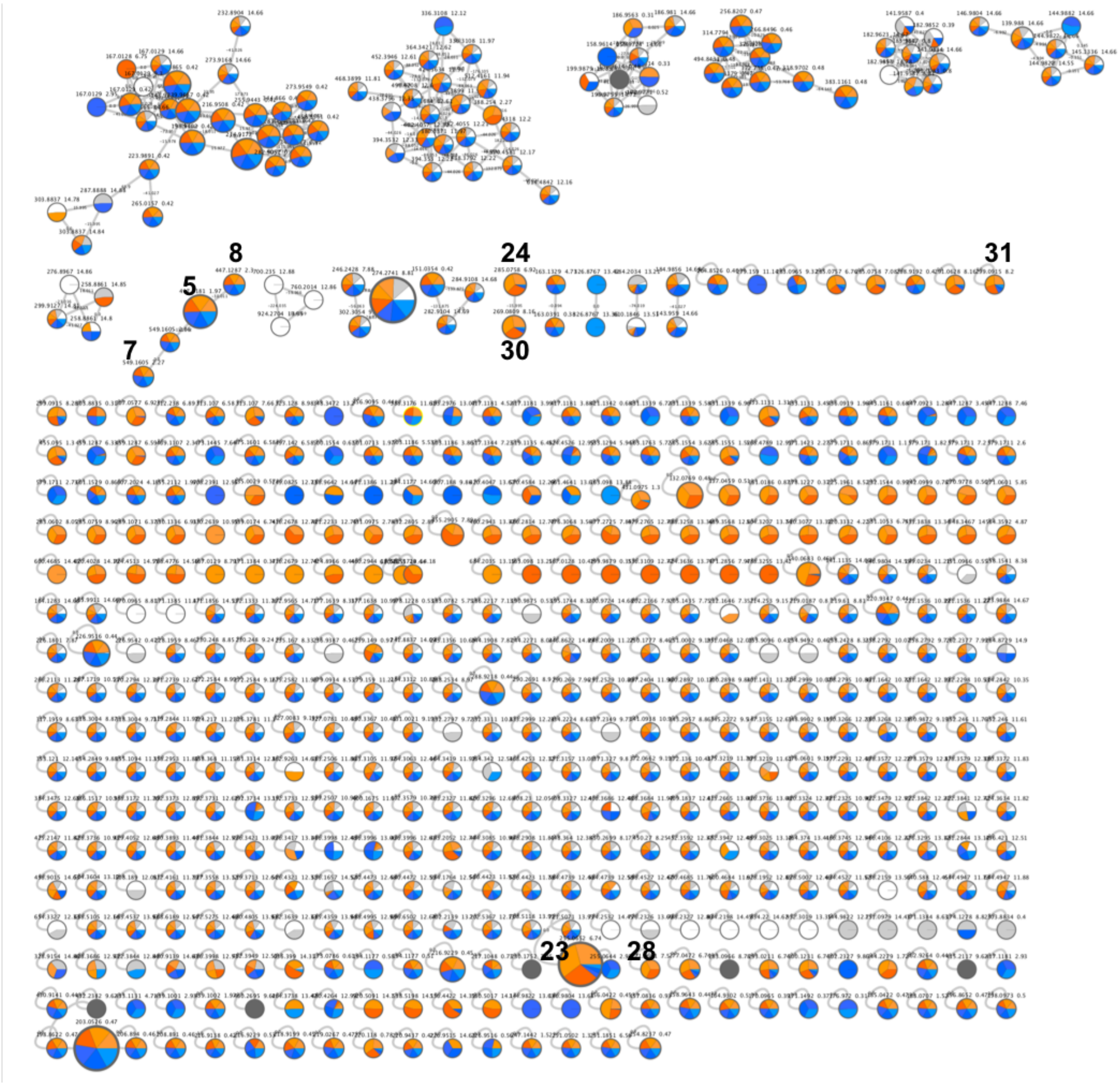

Supplementary Figure S2. Feature-based molecular network obtained with the untargeted UHPLC-HRMS/MS analyses of permeation experiment (section 4.8). The data result from the processing of the analyses, comparing PLRE at 200 µg/mL at 0 min (blue tones) and at 100 min (orange tones). Blank solutions are represented in white and grey. Node size represents the peak height intensity in the donor at 100 min. The numbers indicated the peaks detected in the membrane (table 1).

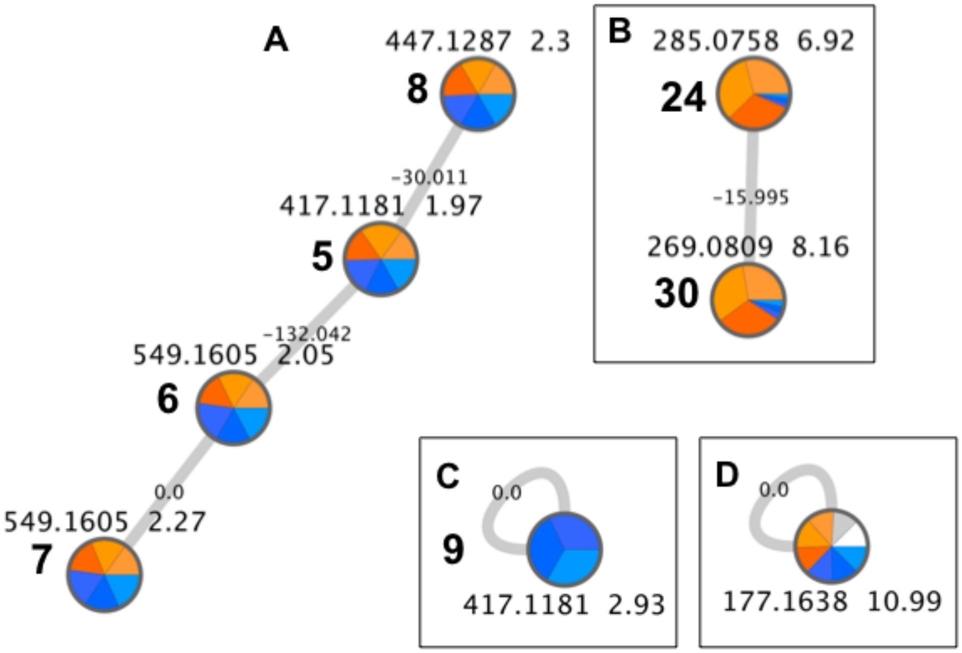

Supplementary Figure S3. Examples of visualization in the FBMN of different behaviors of PLRE constituents in the presence of jejunum. The data result from the processing of untargeted UHPLC-HRMS/MS analyses after the permeation experiment, comparing PLRE at 200 µg/mL at 0 min (blue tones) and at 100 min (orange tones) (section 4.8). Blank solutions are represented in white and grey. Each node is labelled with its *m*/*z* and retention time data, each edge with the *m*/*z* difference between the two connected nodes. Arbitrary node size. A: the peaks are stable, B: the peaks increased in donor compartment, C: the peak disappeared in donor compartment, D: UHPLC-HRMS contamination. The numbers referred to table 1.

2 Semi-quantitative peak ratios from UHPLC-HRMS data processing

Table S1: Semi-quantitative ratio calculated between the average intensity of the peak heights in the acceptor at $T_{100\ min}$ and donor compartments at $T_{0\ min}$ (stock solution) and at $T_{100\ min}$ (equations 1 and 2) (targeted deconvolution):

				Ratio		Acceptor at $T_{100\ min}$				Stock solution at $T_{0\ min}$				Donor at T_{100min}			
**N°**	***m/z* [M+H]+**	**RT[a] (min)**	**Annotation**	**Acc[b]/T_{0min}**	**Acc[b]/$T_{DON100\ min}$**	**Average**	**SD**	**N**	**CV**	**Average**	**SD**	**N**	**CV**	**Average**	**SD**	**N**	**CV**
**5**	417.1180	1.97	puerarin	0.10%	0.10%	2.4E+04	4.1E+03	3	17.0%	2.5E+07	3.3E+05	3	1.3%	2.5E+07	2.5E+06	4	10.0%

[a] Retention time, [b] Acceptor

Table S2: Semi-quantitative ratios calculated between the average intensity of the peak heights in the extracted membranes and donor compartments at $T_{0\ min}$ (stock solution) (equation 3) (targeted deconvolution):

					Extracted membrane				Stock solution at $T_{0\ min}$			
**N°**	**m/z [M+H]+**	**RT[a] (min)**	**Annotation**	**Ratio M[b]/$T_{0\ min}$**	**Average**	**SD**	**N**	**CV**	**Average**	**SD**	**N**	**CV**
**5**	417.1180	1.97	puerarin	0.5%	1.2E+05	1.6E+04	4	13.6%	2.5E+07	3.3E+05	3	1.3%
**7**	549.1605	2.27	6''-xylo-puerarin	0.3%	9.8E+03	1.5E+03	4	15.5%	3.4E+06	2.4E+05	3	6.8%
**8**	447.1286	2.30	3'-methoxypuerarin	0.6%	2.4E+04	4.0E+03	4	16.2%	4.4E+06	2.6E+05	3	5.8%
**23**	255.0652	6.74	daidzein	13.4%	6.1E+05	1.9E+05	4	30.4%	4.6E+06	1.7E+05	3	3.6%
**28**	271.0602	7.51	genistein	116.3%	2.4E+04	2.8E+03	4	11.8%	2.1E+04	7.6E+02	3	3.7%

[a] RT: Retention time, [b] M: extracted membrane

Table S3: Semi-quantitative ratios calculated between the average intensity of the UHPLC-HRMS peak heights of extracted membranes and donor compartments at $T_{100\ min}$ (equation 4) (targeted deconvolution):

				Ratio	Extracted membrane				Donor at T_{100min}			
**N°**	***m/z* [M+H]⁺**	**RT[a] (min)**	**Annotation**	**M[b]/$T_{DON100min}$**	**Average**	**SD**	**N**	**CV**	**Average**	**SD**	**N**	**CV**
**5**	417.1180	1.97	puerarin	0.5%	1.2E+05	1.6E+04	4	13.6%	2.5E+07	2.5E+06	4	10.0%
**7**	549.1605	2.27	6''-xylo-puerarin	0.3%	9.8E+03	1.5E+03	4	15.5%	3.4E+06	2.4E+05	4	6.8%
**8**	447.1286	2.30	3'-methoxypuerarin	0.6%	2.4E+04	4.0E+03	4	16.2%	4.3E+06	1.3E+05	4	3.1%
**23**	255.0652	6.74	daidzein	1.7%	6.1E+05	1.9E+05	4	30.4%	3.5E+07	2.6E+06	4	7.3%
**24**	285.0759	6.93	3'-methoxydaidzein	0.7%	4.1E+04	1.4E+04	4	34.5%	5.8E+06	2.2E+05	4	3.8%
**28**	271.0602	7.51	genistein	3.1%	2.4E+04	2.8E+03	4	11.8%	7.7E+05	3.3E+04	4	4.2%
**30**	269.0810	8.16	formononetin	3.0%	2.4E+05	5.6E+04	4	23.1%	8.0E+06	2.8E+05	4	3.5%
**31**	299.0918	8.20	tithonin	3.7%	2.1E+04	3.6E+03	4	17.6%	5.6E+05	4.0E+04	4	7.2%
**32**	285.0761	8.96	biochanin A	9.0%	1.4E+04	3.6E+03	4	26.2%	1.5E+05	8.0E+03	4	5.2%

[a] RT: Retention time, [b] M: extracted membrane

Table S4: Semi-quantitative ratios calculated between the intensity average of the UHPLC-HRMS peak heights of donor compartments at $T_{100\ min}$ and at T_{0min} (stock solution) (equation 5):

					Stock solution at T_{0min}				Donor at $T_{100\ min}$			
**n°**	***m/z* [M+H]⁺**	**RT[a] (min)**	**Annotation**	**Ratio $T_{DON100\ min}/T_0$ [b]**	**Average**	**SD**	**N**	**CV**	**Average**	**SD**	**N**	**CV**
1	579.1711	0.86	puerarin-6''-O-glucoside	85.9%	5.4E+05	1.4E+04	3	2.6%	4.7E+05	4.4E+04	4	9.5%
2	579.1711	1.10	daidzin-4''-glucoside	0.0%	3.5E+05	2.8E+04	3	8.1%	0	NA	NA	NA
3	433.1131	1.30	3'-hydroxypuerarin	209.2%	1.2E+06	1.9E+04	3	1.6%	2.5E+06	4.0E+04	4	1.6%
4	565.1554	1.52	genistein-8-C-apioside	196.7%	9.3E+04	1.6E+03	3	1.7%	1.8E+05	1.2E+04	4	6.5%
5	417.1183	1.97	puerarin	100.4%	2.5E+07	3.3E+05	3	1.3%	2.5E+07	2.5E+06	4	10.0%
6	549.1605	2.05	mirificin	97.6%	9.8E+05	5.6E+04	3	5.7%	9.5E+05	2.3E+04	4	2.4%
7	549.1604	2.27	6′′-xylo-puerarin	92.6%	3.7E+06	1.4E+05	3	3.7%	3.4E+06	2.4E+05	4	6.8%
8	447.1287	2.30	3'-methoxypuerarin	96.9%	4.4E+06	2.6E+05	3	5.8%	4.3E+06	1.3E+05	4	3.1%
9	417.1181	2.94	daidzin	0.8%	5.9E+06	1.6E+05	3	2.7%	4.7E+04	9.5E+04	4	200.0%
10	447.1287	3.45	3′-methoxydaidzin	0.0%	6.4E+05	1.8E+04	3	2.8%	0	NA	NA	NA
11	433.1131	3.46	genistein-8-C-glucoside	106.2%	1.1E+05	1.7E+03	3	1.5%	1.2E+05	1.7E+03	4	1.5%
12	417.1181	3.89	neopuerarin A	93.4%	1.7E+05	1.8E+03	3	1.1%	1.6E+05	1.2E+04	4	7.3%
13	417.1181	4.00	daidzein-4'-glucoside	20.9%	1.8E+05	5.6E+03	3	3.1%	3.8E+04	7.6E+04	4	200.0%
14	607.2024	4.11	pueroside A	89.7%	1.3E+05	2.0E+03	3	1.5%	1.2E+05	7.3E+03	4	6.1%
15	417.1181	4.53	neopuerarin B	98.0%	1.4E+05	2.3E+03	3	1.7%	1.3E+05	6.0E+03	4	4.5%
16	433.1131	4.71	genistin	0.0%	3.4E+05	1.2E+04	3	3.4%	0	NA	NA	NA
17	503.1186	5.53	6′′-O-malonyl-daidzin	85.0%	1.1E+06	8.7E+04	3	8.0%	9.3E+05	5.7E+04	4	6.1%
18	271.0601	5.86	8-hydroxydaidzein	NA	ND	ND	NA	NA	2.9E+05	1.5E+04	4	5.4%
19	459.1286	6.33	6''-O-acetyldaidzin	6.5%	9.1E+05	5.8E+04	3	6.3%	5.9E+04	7.3E+04	4	123.7%
20	519.1135	6.48	6''-malonylgenistin	83.4%	2.4E+05	3.7E+03	3	1.5%	2.0E+05	1.2E+04	4	6.1%
21	475.1601	6.58	pueroside D	106.2%	1.4E+06	7.5E+04	3	5.3%	1.5E+06	1.5E+05	4	9.9%
22	431.1338	6.73	ononin or isomer	0.0%	2.0E+06	6.2E+04	3	3.0%	0	NA	NA	NA
23	255.0652	6.74	daidzein	774.5%	4.6E+06	1.7E+05	3	3.6%	3.5E+07	2.6E+06	4	7.3%
24	285.0758	6.93	3'-methoxydaidzein	1456.5%	4.0E+05	1.2E+04	3	2.9%	5.8E+06	2.2E+05	4	3.8%
25	431.1338	6.96	ononin or isomer	0.0%	2.1E+05	1.1E+04	3	5.3%	0	NA	NA	NA

26	285.0757	7.08	kakkatin	NA	ND	NA	3	NA	4.8E+05	2.0E+04	4	4.3%
27	447.1288	7.46	sissotrin	0.0%	1.0E+05	3.3E+03	3	3.2%	0	NA	NA	NA
28	271.0601	7.50	genistein	*3736%[td]*	*2.1E+04*	*7.6E+02*	*3*	*3.7%*	7.7E+05	3.3E+04	4	4.2%
29	313.1070	7.66	puerol B	NA	ND	NA	3	NA	1.6E+05	2.1E+04	4	13.3%
30	269.0809	8.16	formononetin	1033.4%	7.7E+05	3.1E+04	3	4.0%	8.0E+06	2.8E+05	4	3.5%
31	299.0915	8.20	tithonin	422.2%	1.3E+05	2.8E+03	3	2.2%	5.6E+05	4.0E+04	4	7.2%
32	285.0759	8.96	biochanin A	NA	ND	NA	3	NA	1.5E+05	8.0E+03	4	5.2%

[a] RT: Retention time, [b] **T_{100}/T_0** ratio between the average intensity of peak height in donor compartment at $T_{100\ min}$ and $T_{0\ min}$

[td]: peak height intensities obtained by targeted deconvolution (td) and verified with LOQ measurement. ND: non detected; NA: non adapted

3 Quantitative results for PLRE

Table S5: Measured concentration in PLRE of five constituents at $T_{0\ min}$ (stock solution) and in the donor compartments at T_{100min}

Substance	**Stock solution at T_{0min}** Concentration ± SD (CV) (n=3)	**Donor at T_{100min}** Concentration ± SD (CV) (n=5)
**Puerarin**	25.724 ± 0.288 µM (1.12%)	29.574 ± 0.997 µM (3.4%)
**Daidzin**	5.035 ± 39.67 µM (0.79%)	*181 ± 94 nM (52.0%)*
**Genistin**	*403.28 ± 5.96 nM (1.48%)*	*22 ±2 nM (9.1%)*
**Daidzein**	*138 ± 3.52 nM (2.54%)*	1.544 µM ± 0.197 (12.8%)
**Genistein**	Trace amount	121 ± 15 nM (12.8%)

Table S6: Measured amount of puerarin (**5**) in PLRE during permeation experiment

Puerarin in PLR	Ratio (nmole/nmole)		Average (nmole)	SD (nmole)	N	CV (%)
**Stock solution (T_{0min})**		-	205.793	2.303	3	1.12
**Donor (T_{100min})**		100.64%	207.103	7.044	5	3.4
**Membrane**	Q_{DEP}[a]	0.22 %	0.455	0.058	5	12.9
**Acceptor(T_{100min})**	Q_{PERM}[b]	0.24 %	0.499	0.054	5	10.8
**Sum**		101.10%	208.057	7.083		

[a] calculated with equation 7, [b] calculated with equation 8

Table S7: Measured amount of daidzin and daidzein in PLRE during permeation experiment

Daidzin/Daidzein in PLRE	Average (nmole)	SD (nmole)	N	CV (%)
**Daidzin**				
**Stock solution (T_{0min})**	40.278	0.317	3	0.79
**Donor (T_{100min})**	1.270	0.660	5	52.0
**Membrane**	nd			
**Acceptor(T_{100min})**	nd			
**Daidzein**				
**Stock solution (T_{0min})**	1.143	0.028	5	2.5
**Donor (T_{100min})**	10.809	1.379	5	12.8
**Membrane**	0.606	0.074	5	12.2
**Acceptor(T_{100min})**	nd			

	Average (%)	SD (%)	N	CV (%)
$Q_{DEP\text{-}EXTRACT}$[A]	5.38	0.96	5	17.9

[a] calculated with equation 9

Table S8: Measured amount of genistin and genistein in PLRE during permeation experiment

Genistin/Genistein in PLRE	Average (nmole)	SD (nmole)	N	CV (%)
**Genistin**				
**Stock solution (T_{0min})**	3.226	0.048	3	1.5
**Donor (T_{100min})**	0.154	0.014	5	9.1
**Membrane**	nd			
**Acceptor(T_{100min})**	nd			
**Genistein**				
**Stock solution (T_{0min})**	1.143	0.028	5	2.54
**Donor (T_{100min})**	0.848	0.108	5	12.8
**Membrane**	0.338	0.031	5	9.2
**Acceptor(T_{100min})**	nd			
**Blank membranes**	0.101	0.014	3	14.2

	Average (%)	SD (%)	N	CV (%)
Genistein $Q_{DEP\text{-}EXTRACT}^{a,b}$	21.63	3.17	5	14.6

[a] Calculated with equation 9, [b] Calculated by subtracting the average amount in the blank membranes.

4 Quantitative results for puerarin and daidzin standard

Table S9: Quantification results with the standard puerarin

Puerarin standard	Ratio (nmole/nmole)		Average (nmole)	SD (nmole)	N	CV (%)
**Stock solution (T_{0min})**		-	765.254	5.590	3	0.75
**Donor (T_{100min})**		90.74 %	729.921	14.484	4	2.0
**Membrane**	Q_{DEP}[a]	0.47 %	3.813	0.540	4	14.2
**Acceptor(T_{100min})**	Q_{PERM}[b]	0.11 %	0.924	0.098	4	10.6
**Sum**		91.33 %	734.658	353.441		

[a] calculated with equation 7, [b] calculated with equation 8.

Table S10: Quantification results with the standard daidzin, following both daidzin and daidzein

Daidzin standard	Average (nmole)	SD (nmole)	N	CV (%)
**Daidzin**				
**Stock solution (T_{0min})**	811.371	31.181	3	3.8
**Donor (T_{100min})**	341.999	88.216	6	42.15
**Membrane**	nd			
**Acceptor(T_{100min})**	nd			
**Daidzein (metabolite)**				
**Donor (T_{100min})**	572.907	107.626	6	18.8
**Membrane**	5.099	0.625	6	12.3
**Acceptor(T_{100min})**	nd			
**Sum**	**920.005 (113.4%)**			

	Average (%)	SD (%)	N	CV (%)
Q_{DEP} [a] ***(daidzein/daidzin*** **$T_{0\ min}$)**	0.63	0.08	6	12.2
Metabolite Formation Index (MFI) [b]	71.2	13.3	6	18.7

[a] calculated with equation 7, [b] calculated with equation 10.

5 Data processing of the metabolite profiling

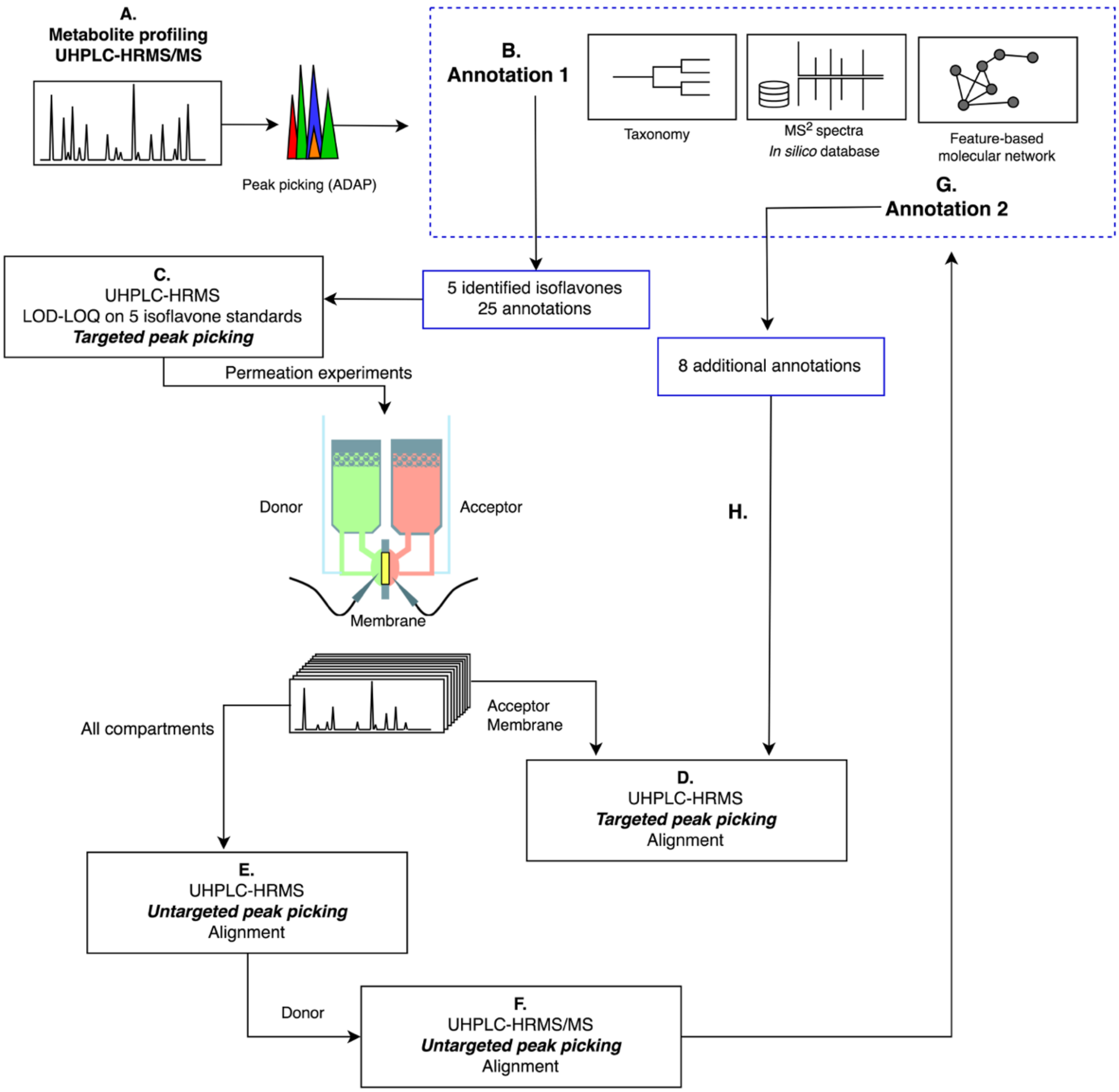

Supplementary Figure S4. Scheme of UHPLC-HRMS and HRMS/MS data processing and annotation strategies established to consider detection limits related to both data acquisition and data processing.

6 Acquisition of the untargeted UHPLC-HRMS/MS metabolite profiling of PLRE

The analyses were performed on an Acquity UPLC system interfaced to an Orbitrap Q-Exactive Focus mass spectrometer (Thermo Scientific, Bremen, Germany), using a heated electrospray ionization (HESI-II) source. Thermo Scientific Xcalibur 2.1 software was employed for instrument control. An Acquity UPLC PDA detector acquired the UV trace from 200 to 500 nm. The full MS analyses were performed in positive mode with a mass range of 150-1500 at a resolution of 35,000 full width at half maximum (FWHM) (at *m/z* 200). Diisooctyl phthalate $C_{24}H_{38}O_4$ $[M+H]^+$ ion (*m/z* 391.28429) was used as an internal lock mass. The optimized HESI-II parameters were the following: source voltage: 3.5 kV (pos); sheath gas flow rate (N2): 52.5 units, auxiliary gas flow rate: 13.75 units, spare gas flow rate: 2.75; capillary temperature: 268.75°C (pos); S-Lens RF Level: 50. The mass analyzer was calibrated according to the manufacturer's directions using a mixture of caffeine, methionine-arginine-phenylalanine-alanine-acetate (MRFA), sodium dodecyl sulfate, sodium taurocholate and Ultramark 1621 in an acetonitrile/methanol/water solution containing 1% acetic acid by direct injection. The data-dependent MS/MS events were performed on the 3 most intense ions detected in full scan MS (Top3 experiment). The MS/MS isolation window width was 1 *m/z*, and the normalized collision energy (NCE) was 35 units. In data-dependent MS/MS experiment, full scans were acquired at a resolution of 35 000 FWHM (at *m/z* 200), and MS/MS scans at a resolution of 17 500 FWHM with a maximum injection time of 50 ms. After being acquired in MS/MS scan, parent ions were placed in a dynamic exclusion list for 2.0 seconds.

7 MZmine parameters for peak picking

Table S11: MZmine parameters for peak-picking

		HRMS/MS		HRMS only
		High	Low	-
1	Mass detection			
	Noise level MS1	7E4	1E4	1E4
	Noise level MS2	1	1	NA
2	Peak detection -> ADAP chromatogram builder			
	Min group size in # of scans	5	5	15
	Group intensity threshold	7E4	1E4	7E4
	Min highest intensity	5E4	2E4	5E4
	m/z tolerance [ppm]	5	5	5
3	Peak detection -> chromatogram deconvolution Wavelets (ADAP)			
	n/z center calculation	MEDIAN		
	S/N threshold	10		
	S/N estimator:	intensity window SN		
	Min feature height	7E4	1E4	7E4
	Coefficient area threshold	100		
	Peak duration range [min]	0.00- 1.50		
	RT wavelet range [min]	0.00 – 0.06		
	m/z range for MS2 scan pairing [Da]	0.025		NA
	RT range for MS2 scan pairing [min]	0.08		NA
4	Isotopic peaks grouper			
	m/z tolerance [ppm]	5		
	Retention time tolerance absolute [min]	0.01		
	Maximum charge	2		
	Representative isotope	Most intense		
5	Filtering -> Duplicate peak filter			
	m/z tolerance [ppm]	1		
	Retention time tolerance absolute [min]	0.01		
6	Alignment -> JOIN			
	m/z tolerance [ppm]	5		
	Weight for m/z [Da]	1		
	Retention time tolerance absolute [min]	0.02		
	Weight for RT	1		
	Require same charge state	☑		
	Compare isotope pattern ->	5 ppm<br>1E3<br>Min score 70%		5 ppm<br>1E2<br>Min score 70%
9	Identification -> Custom Database, adduct search, complex search			
	Retention time tolerance absolute [min]	0.01		0.05
	m/z tolerance [ppm]	5		
	Max complex/adduct peak height	10000%		
11	Filtering -> peak list rows filter			
	Keep only peaks with MS2 scan (GNPS)	☑		
	Reset the peak number ID	☑		
	Export to .csv			
	Export to .mgf for GNPS			

8 Acquisition of the quantitative analyses

The quantitative measurements were performed with a UHPLC system connected with a triple quadrupole (TQ) mass spectrometer operating in multiple reaction monitoring (MRM). The system was equipped with an Acquity UPLC system (Waters, Milford, MA, USA) composed of a binary solvent manager (Class I), a sample manager FL (Class I), and a column manager. This UHPLC system was connected with a triple quadrupole (TQ) mass spectrometer (Waters Xevo TQ-S Micro) equipped with Z-spray® electrospray ionization source (ESI) operating in positive mode. Masslynx V4.1 software was employed for data acquisition, data handling, and instrument control. The nebulizing gas was high purity nitrogen, and the collision gas was high purity argon. The capillary voltage of ESI was set at 3.8 kV, desolvation gas temperature at 350°C, and its gas flow at 650 L/h. Multiple reaction monitoring (MRM) were performed for selective detection and quantification. Cone voltage, and collision energies were optimized for each compound by using the embedded module Intellistart. MRM with the following precursor → product(s) m/z and cone and collision voltages were the following: puerarin, 417 → 267 + 297, cone voltage 62 V and collision one at 28 V; daidzin, 417 → 91 + 199 + 255 cone voltage 44 V and collision ones at respectively 70, 44 and 16 V; genistin, 433 → 91 +153 +271, cone voltage 40 V and collision ones at respectively 66, 48, 28 V; daidzein, 255 → 91 + 137 +199, cone voltage 68 V and collision ones at respectively 30, 24, 22 V; genistein, 271 → 91, 153, 215, cone voltage 70 V and collision ones at respectively 36, 28, 22 V. The signals were integrated by the Targetlynx module included in Masslynx.
